# The Natural Product Andrographolide Ameliorates Calcific Aortic Valve Disease by Regulating the Proliferation of Valve Interstitial Cells *via* the MAPK-ERK Pathway

**DOI:** 10.3389/fphar.2022.871748

**Published:** 2022-04-29

**Authors:** Yuming Huang, Ming Liu, Chungeng Liu, Nianguo Dong, Liang Chen

**Affiliations:** ^1^ Department of Thoracic Surgery, The First Affiliated Hospital of Nanjing Medical University, Nanjing, China; ^2^ Department of Cardiovascular Surgery, Union Hospital, Tongji Medical College, Huazhong University of Science and Technology, Wuhan, China

**Keywords:** CAVD, andrographolide, cell proliferation, valve interstitial cell, MAPK /ERK2 pathway

## Abstract

Calcific aortic valve disease (CAVD) is an active pathobiological process that involves fibrosis and calcification of aortic valve leaflets, thereby causing cardiac hemodynamic changes and eventually heart failure. Cell proliferation changes at the initial stage of CAVD are an important target for pharmaceutical intervention. This study aimed to investigate whether andrographolide (AGP) could inhibit the proliferation of valve interstitial cells (VICs) *in vitro* and *in vivo* to delay the process of CAVD. Cell proliferative factors were tested in both healthy and CAVD aortic valve samples. Cell cycle, cell growth, and calcification of VICs were assessed using flow cytometry, CCK8 assay, EdU staining, and Alizarin Red S staining. The expression of cell proliferative factors and osteogenic factors were quantified by qRT-PCR or immunofluorescence staining. The interaction between AGP and ERK (extracellular regulated protein kinases) was detected by molecular docking. In addition, a high-fat diet-fed animal model was used to verify the effect of AGP on CAVD *in vivo*. In conclusion, we found that AGP ameliorates aortic valve incrassation by inhibiting cell proliferation via the MAPK-ERK signaling pathway. Therefore, AGP is a promising drug that prevents the occurrence of CAVD via regulating cell proliferation.

## Introduction

Calcific aortic valve disease (CAVD) primarily occurs in octogenarians, has high morbidity and mortality rates, and is extremely difficult to treat (Otto and Prendergast, 2014). To date, there is no pharmacological treatment for CAVD and surgery is the only effective cure. Therefore, the effective pharmacological treatment of CAVD remains a research focus (Hutcheson et al., 2014). Based on the histological features, CAVD is divided into two stages: fibrosis and biomineralization. In the initial stage, significant fibrous thickening of the valve interstitium due to a large number of proliferating cells leads to a decrease in leaflet elasticity and an increase in stiffness. Changes in the physical properties of valve leaflets lead to valve dysfunction, resulting in altered cardiac hemodynamics and CAVD-related pathological changes (Mathieu and Boulanger, 2014; Xu et al., 2020).

Andrographolide (AGP) is a natural terpenoid extracted from the traditional Chinese herbal plant *Andrographis paniculata*. AGP has anti-inflammatory, anti-atherosclerotic, and antitumorigenic activities (Yang et al., 2019). Our previous study indicated that AGP could attenuate the ossification of valve interstitial cells (VICs) by inhibiting the inflammatory response (Huang et al., 2020). In addition, the study by Wang *et al.* reported that AGP could regulate the metabolic function of VICs (Wang et al., 2021a).

In the present study, we evaluated whether AGP could attenuate the abnormal proliferation of VICs during the process of aortic valve calcification. This study is an extension of our previous study, which explored the regulatory function of AGP on the proliferation of VICs and the underlying mechanism thereof. Our research indicated that AGP could inhibit the proliferation of VICs via the MAPK-ERK signaling pathway and ameliorate mice aortic valve incrassation induced by *in vivo* high-fat feeding.

## Materials and Methods

### Cell Culture and Treatments

This study was approved by the Ethics Committee of Wuhan Union Hospital, Tongji Medical College, Huazhong University of Science and Technology in Wuhan, China. Patients provided signed informed consent. Calcific aortic valves samples were obtained from CAVD patients undergoing aortic valve replacement surgery, while control healthy aortic valves were obtained from patients undergoing Bentall surgery. Aortic valves were digested in 2 mg/ml type I collagenase (Sigma-Aldrich, Saint Louis, MO) for 6 h at 37°C, 5% CO_2_ after washing with phosphate-buffered saline (PBS). Undigested tissues were removed using the 70 μm nylon cell filters; cells were then seeded in high glucose Dulbecco’s modified Eagle’s medium (DMEM) supplemented with 10% fetal bovine serum (FBS, Gibco Laboratories, Gaithersburg, MD) for primary cultures. We used the osteogenic medium (OM) to induce the osteogenic differentiation of VICs, and AGP was added to inhibit this process. Every experiment was repeated three times for each aortic valve sample (Xu et al., 2019; Liu et al., 2020; Zhou et al., 2020).

### Western Blotting and Immunofluorescence

Western blotting: cell samples were extracted, quantified, and then boiled at 95 °C for min. The protein sample was separated on an 8% sodium dodecyl sulfate-polyacrylamide electrophoresis gel and then transferred on a polyvinylidene fluoride membrane. Finally, primary antibodies were incubated overnight at 4 °C, followed by the corresponding secondary antibodies for protein expression visualization.

Immunofluorescence: Cell samples were seeded on 24-well confocal culture plates at a density of 10,000 cells/well. Samples were washed twice with PBS, fixed in 4% paraformaldehyde for 15 min, and then washed again with PBS. Cells were permeabilized with 0.2% Triton X-100 for 10 min and blocked for 30 min with goat serum albumin (Boster, Wuhan, China). Then, it was incubated with primary antibodies at 4°C overnight, followed by incubating the fluorescent secondary antibody in the dark for 40 min at room temperature. Finally, the samples were washed twice with PBS and incubated with DAPI (Biofroxx GmbH, Einhausen, Germany) for a few minutes to stain the nuclei; then, they were imaged on the Axio Observer Z1 microscope (Zeiss, Oberkochen, Germany). The following antibodies were used: Runx2 (CST: 8486s, 1:1000), ALP (R&D systems: MAB29092, 1:500), CDK1 (Proteintech: 19532-1-AP, 1:500), KI67 (Proteintech: 27309-1-AP, 1:200), anti-phospho-p38 (Thr180/Tyr182) (CST: 4511, 1:1000), anti-P38 (CST:8690, 1:1000), anti-phospho-p44/42 mitogen-activated protein kinase (Erk) (Thr202/Tyr204) (CST: 4377, 1:1000), anti-Erk (CST: 4695, 1:1000), and E2F1 (Abcam: ab179445, 1:1000).

### RNA Extraction and qPCR Analysis

Total cell RNA was extracted with Trizol reagent (Invitrogen, Carlsbad, CA). The real-time polymerase chain reaction (PCR) was performed on a StepOne Plus thermal cycler (Applied Biosystems, Foster City, CA) using a 2x SYBR Green qPCR Master Mix (High ROX) (Bimake, Houston, TX) following the manufacturer’s guide. All the primers were referenced from the previous study (Xu et al., 2019). The final data were analyzed by the 2-^ΔΔ*c*^_t_ method. The primers used were as follows: CDK1 (F: 5′-TCC​TCC​AGG​GGA​TTG​TGT​TTT-3′; R: 5′-GCC​AGT​TTG​ATT​GTT​CCT​TTG​TC-3′), E2F1 (F: 5′-ACTTT GGTCTCGAGGAGGGT-3′; R: 5′-TGC​TAT​TCC​AAC​GAG​GCA​GG-3′), and MKI67 (F: 5′-GCC​CCT​GGA​AGA​TTA​TGG​TGG-3′; R: 5′-GGG​TTC​TGA​CTG​GTT​GTG​GTT​GT-3′).

### FACS for Cell Cycle Cell Viability Assay

VICs (passage 3) were cultured in 60 mm dishes under different treatments. Then, the samples were trypsinized and re-suspended in PBS at 5*10^5^/ml, followed by fixation in 70% precooled ethanol overnight at 4°C, centrifugation, washing, and staining with PI/RNase staining buffer (BD Biosciences) for 30 min at 4°C. Cell counts at different phases of the cell cycle were analyzed by flow cytometry (FCM) as previously described (Huang et al., 2019).

### Cell Viability Assay

Cell viability was assessed using the Cell Counting Kit-8 (CCK-8) assay (Bimake.com, Houston, TX, United States) according to the manufacturer’s instructions. The cells were seeded at a density of 5,000 cells/well in 24-well plates and cultured for 6 days under different treatments. At the end of each time interval, the cell samples were washed with PBS and incubated with a serum-free medium containing 10% CCK-8 reagent for 4 h at 37°C, 5% CO_2_, and 10 µl aliquots were pipetted into a 96-well plate and measured at 450 nm using an enzyme-labeling instrument (Thermo Fisher Scientific).

### EdU Assay

Cell proliferation ability was tested by EdU assay (Ribobio bio, Guangzhou, China) according to the manufacturer’s instructions. The cells were seeded at a density of 10,000 cells/well in 12-well plates. After different treatments, VICs were cultured in a medium containing Edu for 2 h at 37°C, 5% CO_2_. The cell samples were fixed with 4% paraformaldehyde for 15 min then washed with PBS several times, stained with apollo staining solution for 30 min, and observed under the fluorescence microscope.

### Molecular Docking

The andrographolide (AGP) compound structure is downloaded from the PubChem database (https://pubchem.ncbi.nlm.nih.gov/). The three-dimensional structure of Erk-2 (PDB ID: 4H3Q) is downloaded from the RCSB Protein Data Bank (www.rcsb.org). Autodock vina 1.1.2 was used for semi-flexible docking. Briefly, the coordinates and box size of the Vina molecule docking were determined, and then the parameter exhaustiveness was set as 20. Except for special instructions, other parameters adopt default values. The best affinity conformation with the lowest docking binding energy is selected as the final docking conformation. Pymol software was used for image construction.

### Calcification Analysis

Cells were seeded into 12-well plates cultured in the OM (contained: 10 mM β-glycerophosphate, 100 nM dexamethasone, 50 μg/ml vitamin C, 2% FBS, 100 IU/ml penicillin/streptomycin) with or without AGP for 21 days. The degree of cell calcification was measured by Alizarin Red S stain based on the previous study (Huang et al., 2019,^2020^ ).

### Animal Model

All animals used in this research were purchased from the Experimental Animal Center of Tongji Medical College, Huazhong University of Science and Technology (Wuhan, China). The ApoE^−/-^ mice were fed a high-fat and high-cholesterol diet (42% fat, 0.25% cholesterol) for 24 weeks to induce aortic valves calcification. To treat CAVD, the disease model mice were treated with AGP at a dose of 10 mg/kg by gavage from 16 to 24 weeks. Then, the heart samples of mice were collected and histologically sectioned to isolate the aortic valves. HE staining was used to evaluate the thickness of the valves. Runx2 expression was analyzed by immunofluorescence staining *in vivo*. The incrassation and Runx2 intensity were measured in the same way as in our previous study (Wang et al., 2021a).

### Statistical Analysis

All experimental data were analyzed and expressed as the mean ± standard deviation (SD). Statistical comparisons were made by the analysis of variance to evaluate differences among different groups. The *p*-value less than 0.05 was considered statistically significant.

## Results

### Increased Proliferation of VICs in CAVD

**FIGURE 1:**
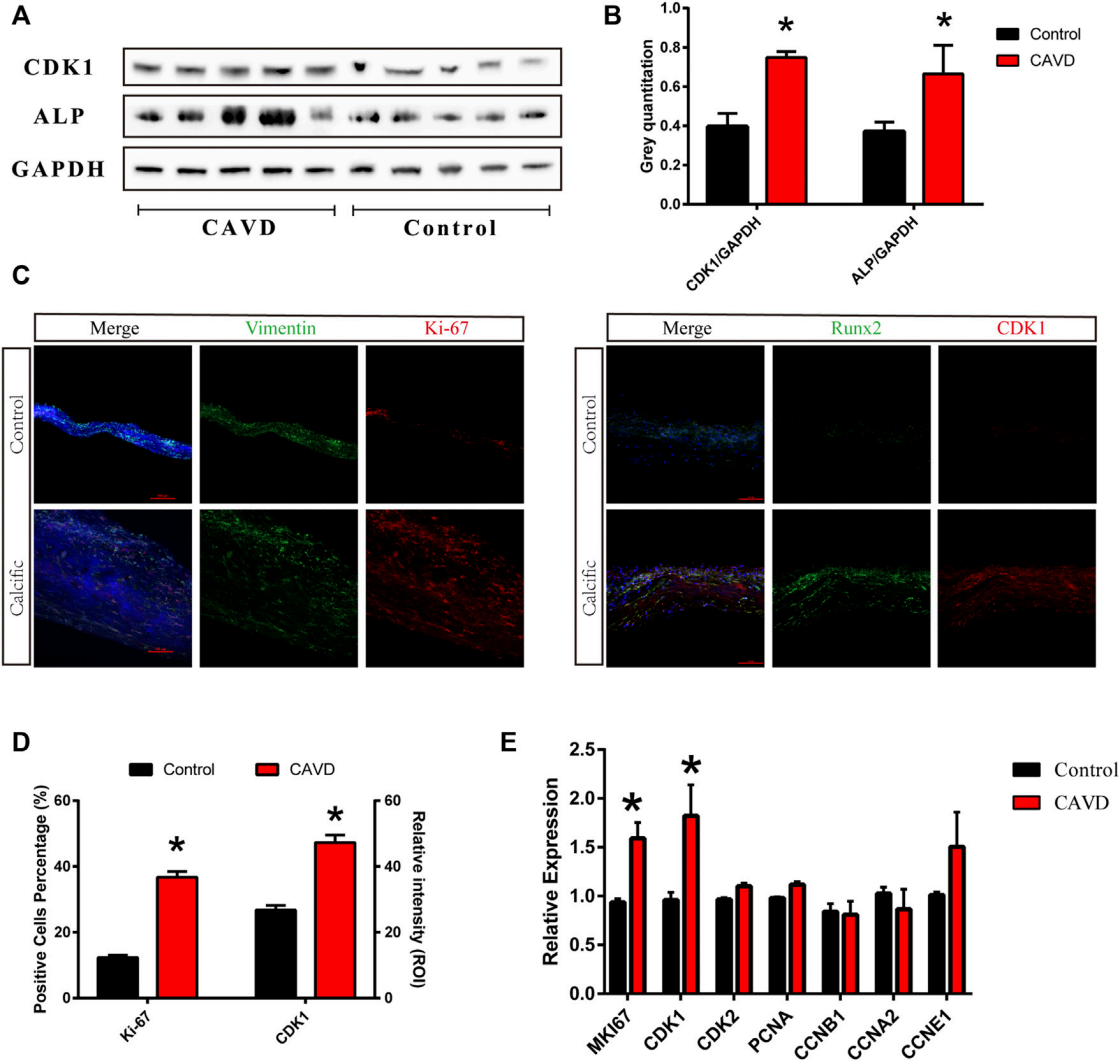
Analysis of the control and CAVD aortic valve samples (*n* = 5 and 5, respectively). **(A)** Western blotting for CDK1 and ALP proteins on valve tissues. **(B)** Semi-quantitative analysis of protein expression. **(C)** Immunofluorescence staining of CDK1 and Ki-67 on valve tissues. **(D)** Semi-quantitative analysis of fluorescence intensity. **(E)** PCR test for cell proliferation genes on valve tissues. (*) *p* < 0.05 indicates a significant difference.

We performed western blotting and immunofluorescence staining on aortic valve tissues to detect the proliferation of VICs in aortic valves from the control and CAVD groups. The cell proliferation-related factors, cyclin-dependent kinase 1 (CDK1), and Ki-67 were upregulated in the CAVD group compared to the control group (Figure 1A,C). The expressions of ALP and CDK1 proteins were significantly increased in the CAVD group compared to the control group (*p < 0.05; Figure 1B). The results of immunofluorescence staining were consistent with those of western blotting (*p < 0.05; Figure 1D). Furthermore, cell proliferation-related genes were tested via real-time PCR; CDK1 and MKI67 genes were significantly upregulated in the CAVD group compared to the control group (*p < 0.05; Figure 1E). Briefly, the proliferation of VICs was significantly increased due to the pathophysiological changes in CAVD.

### AGP Inhibits the Proliferation of VICs *in vitro*

**FIGURE 2:**
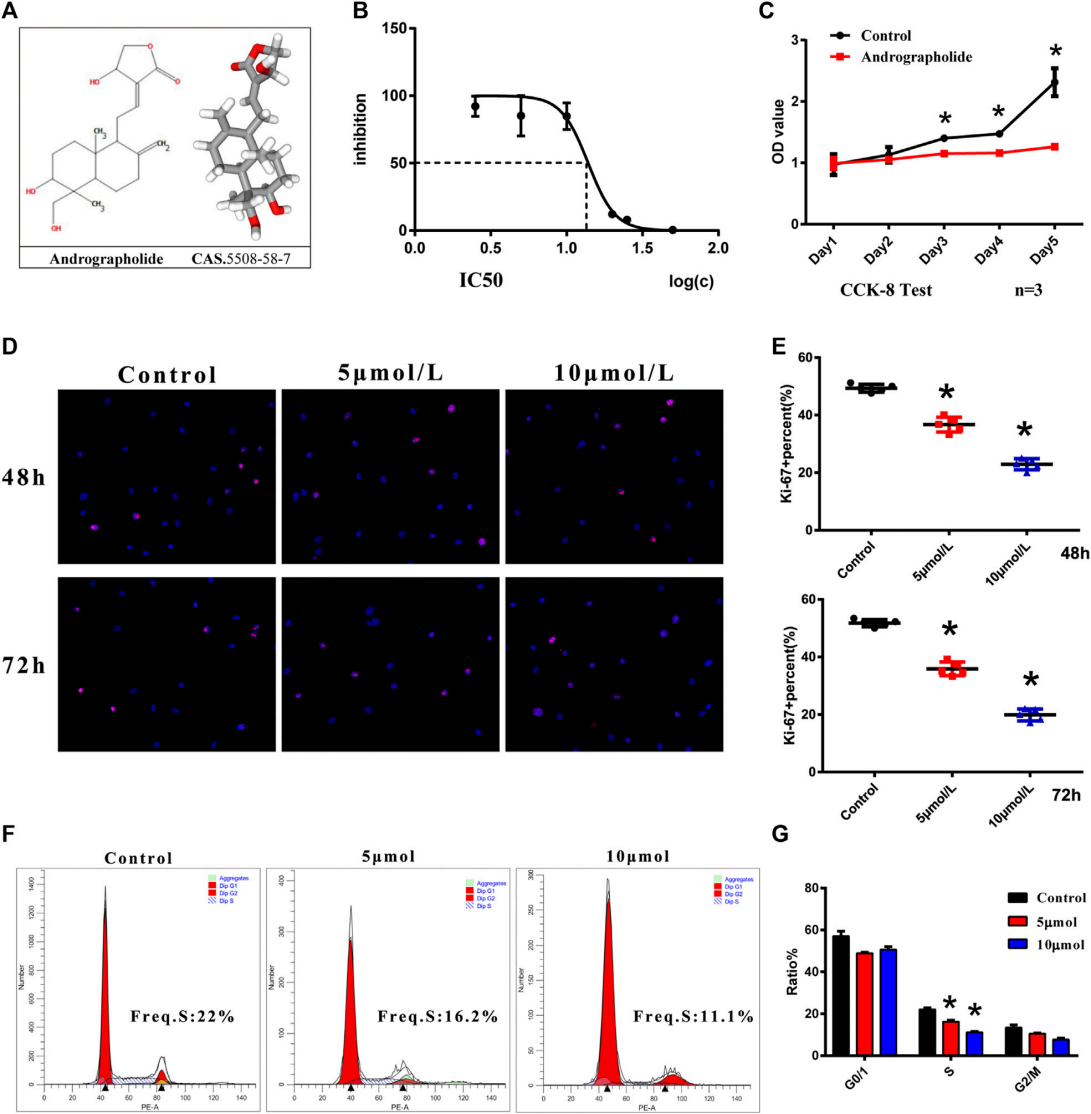
Cell viability and proliferation of VICs with AGP treatment. **(A)** Two- and three-dimensional molecular structure of AGP. **(B)** Cytotoxicity of AGP on VICs. **(C)** Cell proliferation curve after AGP treatment (10 μM) for 5 days compared to the controls (*n* = 3). **(D)** Ki-67 staining of VICs with AGP treatment (red). **(E)** Percentage of Ki-67 positive cells. (*) *p* < 0.05 (vs control) indicates a significant difference. **(F)** Flow cytometry of cell cycle analysis of VICs after AGP treatment (10 μmol/L) for 72 h. **(G)** S, G0/G1, and G2/M phase cells were counted and compared. (*) *p* < 0.05 (vs control) indicates a significant difference (*n* = 4).

The 2D and 3D structures of AGP are shown in Figure 2A. The toxicity of AGP on VICs was assessed by determining the IC50 values, which showed that AGP has significant cytotoxicity at concentrations >10 mΜ (Figure 2B). The results indicated that 10 μΜ is the suitable AGP concentration for our experiments. In addition, the CCK-8 assay was used to analyze cell proliferation after treatment with AGP for 5 days. The results indicated that the cell viability of the AGP group significantly decreased starting from day 3 when compared with the control group (*p < 0.05; Figure 2C). Ki-67 staining indicated that cell proliferation was significantly inhibited after treatments with 5 and 10 μmol/L AGP for 48 and 72 h, respectively (*p < 0.05; Figure 2D,E). Correspondingly, cell cycle analysis showed that the numbers of VICs involved in the S-phase were decreased when cultured in 5 and 10 μM AGP medium for 72 h (*p < 0.05; Figure 2F,G).

### AGP Regulates Osteogenic-Medium-Induced Proliferative Gene/Protein Expression

**FIGURE 3:**
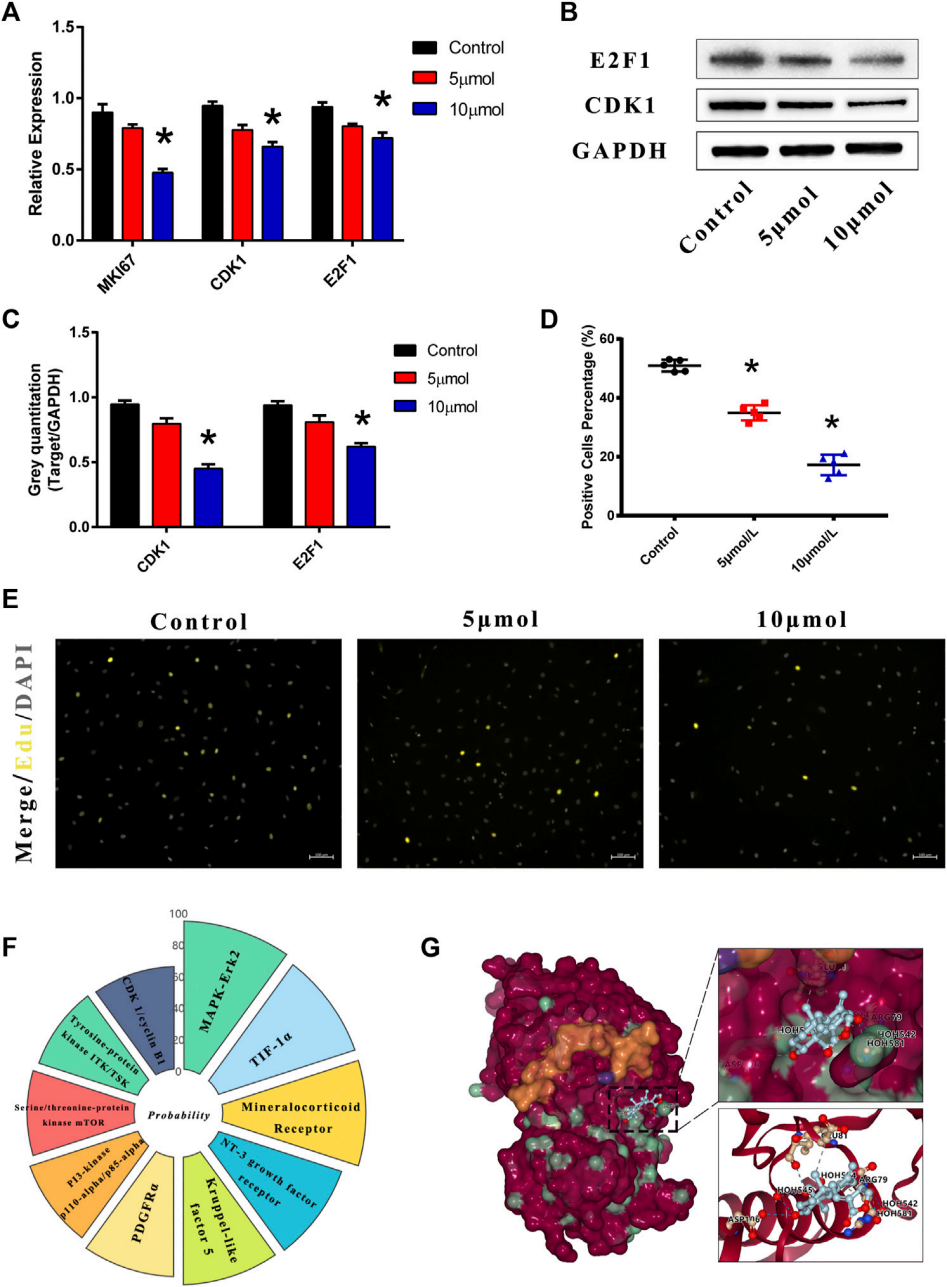
AGP downregulated the cell proliferation genes and protein expression levels. Predicted molecular targets and docking of AGP. **(A)** AGP (5 and 10 μmol/L) inhibited gene expression levels of *CDK1*, *E2F1*, and *MKI67*. **(B)** AGP (5 and 10 μmol/L) inhibited protein expression levels of *CDK1* and *E2F1*. **(C)** Analysis of protein expression levels of CDK1 and E2F1. Data are collected by gray semi-quantification and compared to GAPDH. (*) *p* < 0.05 (vs control) indicates a significant difference. **(D)** Percentage of EdU positive cells. (*) *p* < 0.05 (vs control) indicates significant difference. **(E)** Fluorescence staining of EdU assay in VICs under AGP conditions (yellow). **(F)** Nightingale rose of probabilities in the top 10 targets from the online tool “SwissTargetPrediction.” **(G)** ERK protein was selected for further molecular docking analysis. AGP successfully accessed the pocket structure of the protein molecule.

VICs were treated with AGP for 2 h at doses of 5 and 10 μmol/L. Compared to the control group, expression levels of cell proliferative genes were inhibited by AGP in VICs (Figure 3A). AGP significantly downregulated the mRNA levels of MKI67 (*p < 0.05), CDK1 (*p < 0.05), and E2F1 (*p < 0.05). Similar to the gene expression after treatment, AGP inhibited the CDK1 protein synthesis and downregulated E2F1 expression (*p < 0.05; Figure 3B,C). Based on the immunofluorescence staining of the EdU assay (Figure 3E), 5 and 10 μmol/L AGP inhibited cell proliferation activity relative to the control group, consistent with the levels of gene and protein expression (*p < 0.05; Figure 3D). SwissTarget prediction provided more than 10 potential molecular targets for AGP (Figure 3F), and Erk protein was selected for further molecular docking analysis, based on the previous literature on AGP (Figure 3G). The results indicated that AGP successfully bound to ERK (extracellular regulated protein kinases) using the residues HOH581, HOH542, HOH524, and HOH545 (red dot), via hydrogen bond interactions and residues ASP106, ARG79, GLU81, and ILE83.

### AGP Inhibits the Proliferation of VICs via the MAPK-ERK Pathway

**FIGURE 4:**
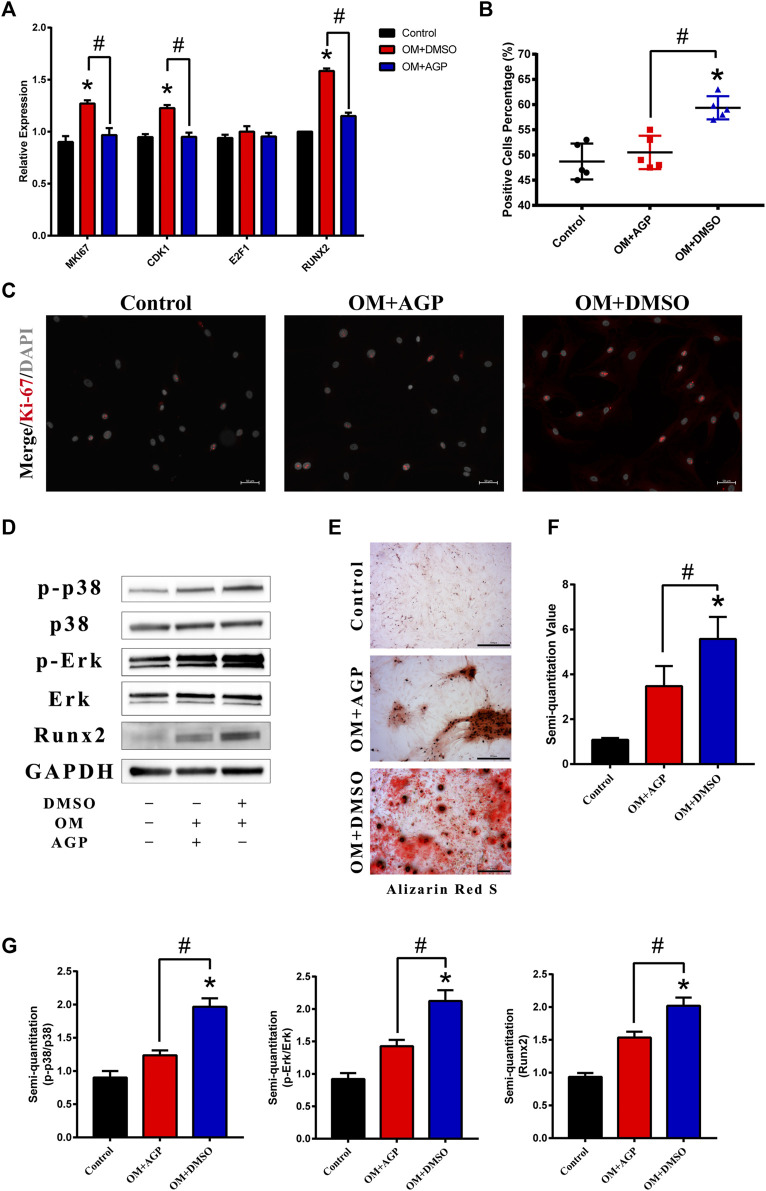
AGP inhibited cell proliferation induced by OM via the MAPK-ERK pathway. **(A)** PCR test for MKI67, CDK1, E2F1, and Runx2 of VICs stimulated by OM with or without AGP for 48 h. **(B)** Percentage of Ki-67 positive cells. (*) *p* < 0.05 (vs control) and (#) *p* < 0.05 (vs OM + DMSO) indicate significant difference. **(C)** Immunofluorescence staining of MKI67 (red). **(D)** Western blotting for Runx2, phosphorylated ERK (p-ERK), total ERK, phosphorylated p38 (p-p38), and total p38 of the cells stimulated by OM with or without AGP for 72 h. **(G)** Statistical analysis of protein expression of Runx2, p38, and ERK according to the gray semi-quantification compared to the GAPDH expression. (*) *p* < 0.05 (vs control) and (#) *p* < 0.05 (vs OM + DMSO) indicate significant difference. **(E,F)** Alizarin Red S staining and semi-quantification of VICs under different cultures: control, OM + DMSO, and OM + AGP (*) *p* < 0.05 (vs control). and (#) *p* < 0.05 (vs OM + DMSO) indicate significant difference (*n* = 3).

Based on the target analysis above, we focused on the MAPK-ERK signaling pathway. The osteogenesis-specific gene *Runx2* and cell proliferative genes *CDK1* and *MKI67* were tested in VICs under an OM culture with or without AGP for 48 h. The results demonstrated that the OM culture significantly increased the expression of *CDK1, MKI67*, and *Runx2*. However, after the addition of AGP to the OM culture, *CDK1, MKI67,* and *Runx2* were significantly downregulated (* vs control group *p* < 0.05; # vs OM + DMSO group *p* < 0.05; Figure 4A). The results of the Ki-67 staining were consistent with the PCR results (Figure 4C). AGP significantly inhibited the OM-induced cell proliferative activity (* vs control group *p* < 0.05; # vs OM + DMSO group *p* < 0.05; Figure 4B). We detected the protein expression levels of phosphorylated ERK and P38 after OM treatment with or without AGP and found that AGP effectively reversed the OM-induced upregulation of phosphorylated ERK and P38 protein expression (Figure 4D,G). After being cultured in OM for 21 days, VICs were tested for calcification using Alizarin Red S staining. OM + DMSO and OM + AGP groups stained positively for Alizarin Red S, showing significant differences compared to the control group (Figure 4E). Semi-quantitation of stain intensity indicated an approximately 1-fold decrease in the OM + AGP group compared to the OM + DMSO group (* vs control group *p* < 0.05; # vs OM + DMSO group *p* < 0.05; Figure 4F).

### AGP Ameliorates Valve Thickening in High-Fat Diet-Fed Mice

**FIGURE 5:**
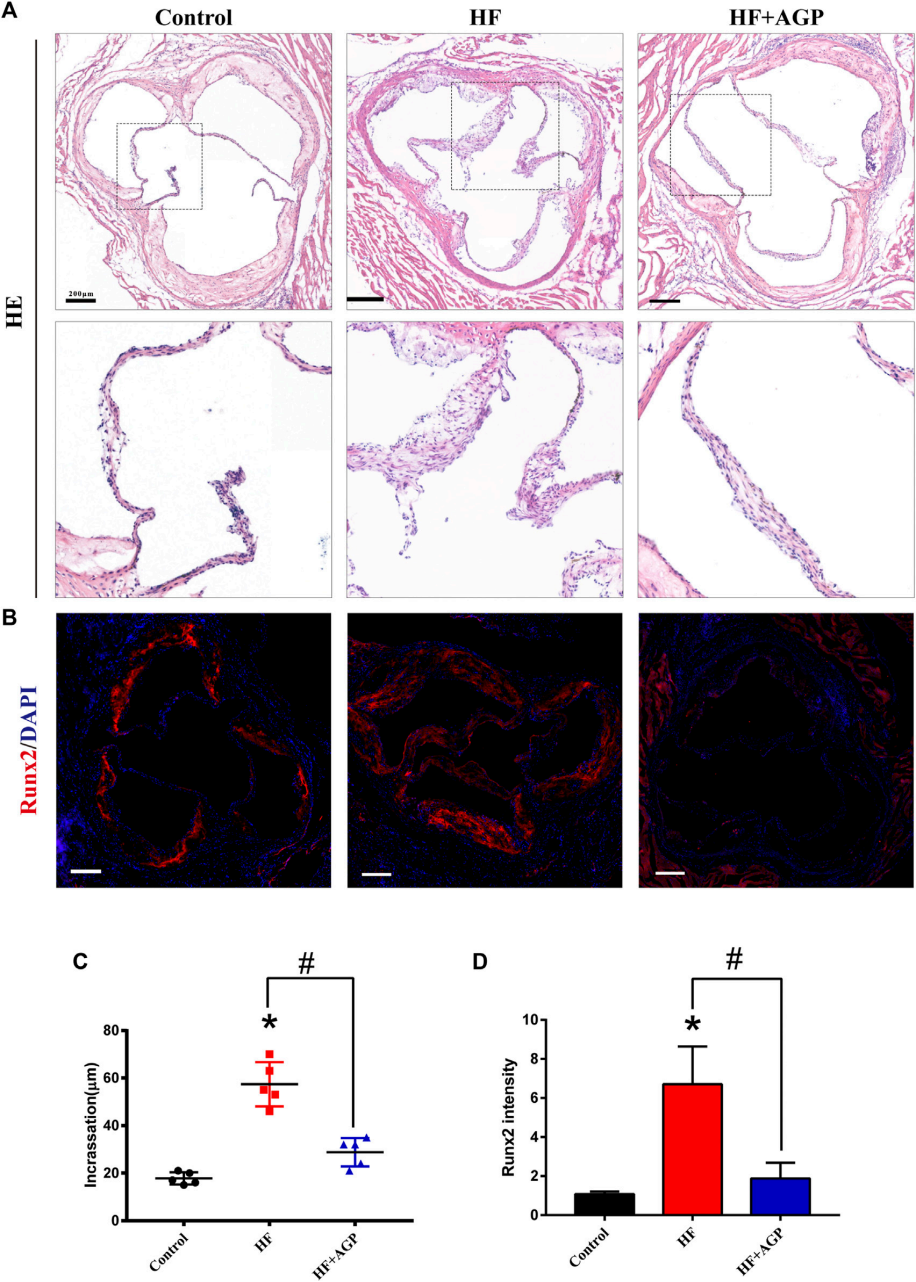
AGP delayed aortic valve disease in high-fat diet-fed (HF) mice. **(A)** Hematoxylin and eosin (HE) staining of HF mice aortic valves with or without AGP treatment (bar = 100 μm). **(B)** Runx2 immunofluorescence staining of HF mice aortic valves with or without AGP treatment (bar = 100 μm). **(C)** Statistical analysis of mice aortic valve calcific incrassation. **(D)** Statistical analysis of mice aortic valve Runx2 fluorescence intensity. (*) *p* < 0.05 (vs control group) and (#) *p* < 0.05 (vs HF group) indicate a significant difference.

We used the high-fat diet-fed animal model to verify the effect of AGP on CAVD *in vivo*. Hematoxylin and eosin (HE) staining and Runx2 immunofluorescence staining were performed on the aortic valve samples of mice (Figure 5A,B). The results demonstrated that the aortic valves of high-fat diet-fed mice were significantly thickened, while AGP feeding ameliorated the aortic valve incrassation (* vs control group *p* < 0.05; # vs HF (high-fat diet) group *p* < 0.05; Figure 5C). Immunofluorescence staining showed that AGP significantly reduced the *Runx2* expressions induced by high-fat diet-feeding *in vivo* (* vs control group *p* < 0.05; # vs HF group *p* < 0.05; Figure 5D).

## Discussion

In this study, AGP was used as a negative regulator of CAVD and we found that AGP could delay CAVD progression. Mechanistically, AGP could act as a modulator of the MAPK-ERK signaling pathway to inhibit the cell proliferation activity of VICs and ameliorate the incrassation of aortic valves. Both *in vivo* and *in vitro* experiments supported our conclusions.

In this study, we performed western blotting, immunofluorescence staining, and real-time PCR test on aortic valve tissues. We found that the cell proliferative factors were upregulated due to the pathophysiological changes in CAVD. In the current research, CAVD was divided into two different stages according to the histological features: fibrosis and biomineralization (Mathieu and Boulanger, 2014; Kostyunin et al., 2019). Histological examination revealed significant fibrosis and thickening accompanied by numerous proliferating cells, resulting in decreased elasticity and increased hardness of the valve leaflets (Jenkins et al., 2020). The cell proliferative factors in aortic valves are upregulated by growth factors, such as transforming growth factors, fibroblast growth factors, or epidermal growth factors, which are abnormally increased in CAVD (Wu et al., 2017; Cho et al., 2018). Therefore, drugs that inhibit early valve calcification according to this feature may be effective treatments for CAVD (Wang et al., 2021b). AGP is a natural compound that we used in our previous study and found that it inhibits the osteogenic transition of VICs (Huang et al., 2020). In this study, we verified that AGP inhibits cell proliferation activity as well. This new finding suggests that AGP may play a role in the early lesions of CAVD, thereby expanding the time window of use of this drug. The osteogenic transition of VICs in CAVD is the most important and widely studied target for the development of pharmacological treatments (Lindman et al., 2016). However, the identification of cell proliferation as a drug target is a breakthrough for drug development. The causal relationship between cell proliferation and osteogenic transition remains controversial (Hulin et al., 2019). Some studies reported that the cell cycle changed during the osteogenic transition, and stimulation of osteogenic induction promoted cell proliferation in the initial stage but cell apoptosis dominated in the terminal stages. In contrast, some studies suggested that cell proliferation occurred when VICs were transformed into osteoblasts in the terminal stages (Rutkovskiy et al., 2017).

In our research, we found that AGP could inhibit cell proliferation via the MAPK-ERK signaling pathway, which may play an important role in cell proliferation and osteogenic transition. In our previous study, we identified AGP as a modulator of NF-κB pathway activation in VICs through transcriptome sequencing analysis. A comprehensive analysis of the relationship between the two signaling pathways suggests that when the MAPK-ERK signaling pathway is inhibited, the NF-KB signaling pathway is also affected (Ott et al., 2014; Zielinska and Katanaev, 2019). We performed target prediction using molecular docking simulations and found ERK2 protein as a promising drug target (Ferreira et al., 2015). However, further research is needed to determine the mechanism underlying the binding of AGP to the ERK protein. We also performed an *in vivo* experiment in mouse disease models to verify our findings. Based on the results of *in vivo* experiments, AGP can effectively delay valve thickening caused by high-fat diet feeding as well as Runx2 factor expression.

Our research had several limitations. Additional research is needed to verify the AGP target of ERK2 protein and the relationship between them. Another limitation was the *in vivo* pharmacokinetics of AGP; some studies suggest that AGP is not readily absorbed. Finally, the specificity and efficacy of AGP for valvular disease need to be determined, which is a major issue in the drug development for valvular disease (Myasoedova et al., 2018).

## Conclusion

AGP inhibited OM-induced cell proliferation and calcification of VICs primarily via the MAPK-ERK signaling pathway. AGP may be a promising therapeutic supplement to prevent and delay the occurrence and progression of CAVD.

## Data Availability

The original contributions presented in the study are included in the article/supplementary materials; further inquiries can be directed to the corresponding authors.
